# Interleukin-29 Enhances Synovial Inflammation and Cartilage Degradation in Osteoarthritis

**DOI:** 10.1155/2016/9631510

**Published:** 2016-06-28

**Authors:** Lingxiao Xu, Qiuyue Peng, Wenhua Xuan, Xiaoke Feng, Xiangqing Kong, Miaojia Zhang, Wenfeng Tan, Meilang Xue, Fang Wang

**Affiliations:** ^1^Department of Rheumatology, The First Affiliated Hospital of Nanjing Medical University, Nanjing 210029, China; ^2^Department of Traditional Chinese Medicine, The First Affiliated Hospital of Nanjing Medical University, Nanjing 210029, China; ^3^Department of Cardiology, The First Affiliated Hospital of Nanjing Medical University, Nanjing 210029, China; ^4^Sutton Arthritis Research Laboratories, University of Sydney at Royal North Shore Hospital, Sydney, NSW, Australia

## Abstract

We have recently shown that IL-29 was an important proinflammatory cytokine in pathogenesis of rheumatoid arthritis (RA). Inflammation also contributes to the pathogenesis of osteoarthritis (OA). The aim of this study was to investigate the effect and mechanism of IL-29 on cytokine production and cartilage degradation in OA. The mRNA levels of IL-29 and its specific receptor IL-28Ra in peripheral blood mononuclear cells (PBMCs) were significantly increased in OA patients when compared to healthy controls (HC). In the serum, IL-29 protein levels were higher in OA patients than those in HC. Immunohistochemistry revealed that both IL-29 and IL-28Ra were dramatically elevated in OA synovium compared to HC; synovial fibroblasts (FLS) and macrophages were the main IL-29-producing cells in OA synovium. Furthermore, recombinant IL-29 augmented the mRNA expression of IL-1*β*, IL-6, IL-8, and matrix-metalloproteinase-3 (MMP-3) in OA FLS and increased cartilage degradation when* ex vivo* OA cartilage explant was coincubated with OA FLS. Finally, in OA FLS, IL-29 dominantly activated MAPK and nuclear factor-*κ*B (NF-*κ*B), but not Jak-STAT and AKT signaling pathway as examined by western blot. In conclusion, IL-29 stimulates inflammation and cartilage degradation by OA FLS, indicating that this cytokine is likely involved in the pathogenesis of OA.

## 1. Introduction

Interleukin-29 (IL-29) is the main cytokine among three members of interferon lambda family (IFN-*λ*s), also known as type III IFNs, including IFN-*λ*1, IFN-*λ*2, and IFN-*λ*3 (or IL-29, IL-28A, and IL-28B, resp.). The activity of IL-29 is determined in part by the distribution and expression of its specific IL-28 receptor alpha chain (IL-28Ra). IL-28Ra is widely expressed by ranges of cells including epithelial cells, hepatocytes, fibroblasts [1, 2], and immune cells, such as plasmacytoid DCs, macrophages, Th17 cells, and NK cells [3–6]. The most studied biological role of IFN-*λ*s is its antiviral activity, but recent investigation starts to uncover a unique role of IFN-*λ*s in and beyond innate antiviral immunity [4, 7]. IL-29 can induce antiproliferative and antitumor properties [8, 9], inhibit IL-13 release in T cells [1], and stimulate IL-6, IL-8, and IL-10 production in macrophages [2] and IL-4 and IL-13 in mast cells [10]. A recent study showed that IL-29 is able to indirectly affect NK cells by regulating IL-12 in macrophages [6]. Moreover, our recent studies have demonstrated that IL-29 levels are higher in peripheral blood mononuclear cells (PBMCs) and serum and synovium from rheumatoid arthritis (RA) patients when compared to healthy individuals [1]. Recombinant IL-29 enhances IL-6, IL-8, and matrix-metalloproteinases (MMPs) production in RA fibroblasts and promotes RA inflammation [1]. IL-29 has also been shown to contribute to other immune diseases, such as systemic lupus erythematosus (SLE) [11, 12], asthma [3], and psoriasis [5]. However, the role of IL-29 in inflammatory diseases remains largely unexplored and whether IL-29 is involved in the pathogenesis of osteoarthritis (OA) is unclear.

OA is one of the most common disorders and has traditionally been classified as a noninflammatory arthritis; however, increasing evidence shows that synovitis and the immune system are also active players in OA development and progression [4]. For examples, OA synovial fibroblasts (FLS) contribute to OA pathogenesis by producing inflammatory cytokines such as tumor necrosis factor-*α* (TNF-*α*), IL-1*β*, and chondrolytic mediators such as MMPs [5]; FLS-derived IL-1*β* and TNF-*α* induce cartilage degradation [13]. In addition, FLS can mediate the innate immune response by responding to both inflammatory cytokines and Toll-like receptor (TLR) ligands [14]. We have shown that IL-29 enhanced RA inflammation and mediated TLR activation in RA FLS [1, 2]. OA and RA share some similarities in pathogenesis, such as chronic inflammation, synovial hyperplasia, articular destruction, and abnormal immune response. We therefore hypothesize that IL-29 contributes to OA pathogenesis. To address this issue, we examined the potential role of IL-29 as a proinflammatory cytokine in OA disease.

This study, for the first time, showed that IL-29 was higher in blood and synovium from OA patients when compared with healthy controls (HC). Recombinant IL-29 increased the expression of proinflammatory cytokine in OA FLS* in vitro* and promoted cartilage degradation in coculture of OA FLS and cartilage explant* ex vivo*. Finally, our studies revealed that the effect of IL-29 on OA FLS was likely mediated by NF-*κ*B and MAPK signaling pathway. Our present data support the hypothesis that IL-29 may contribute to synovial inflammation and cartilage degradation in OA.

## 2. Materials and Methods

### 2.1. Reagents

Recombinant human IL-29 and IL-1*β* were purchased from Peprotech (Rocky Hill, NJ, USA); rabbit anti-human IL-29/IL-28R*α* polyclonal antibody and mouse anti-human CD68 monoclonal antibody were purchased from Abcam (Cambridge, MA, USA); mouse anti-human fibroblast growth factor-basic (FGF-2) monoclonal antibody was purchased from Millipore (Billerica, MA, USA); PE anti-human IL-28R*α* was purchased from BioLegend (San Diego, CA, USA); donkey anti-rabbit IgG-R and goat anti-rabbit IgG/TRITC were purchased from Santa Cruz Biotechnology (Santa Cruz, CA, USA); DyLight*™*488-conjugated AffiniPure donkey anti-mouse IgG, peroxidase-conjugated sheep anti-rabbit secondary antibody, and peroxidase-conjugated sheep anti-mouse secondary antibody were purchased from Jackson Immunoresearch (West Grove, PA, USA); human IL-29 antibody was purchased from R&D Systems (Minneapolis, MN, USA); human IL-29 enzyme-linked immunosorbent assay (ELISA) reagent kits were purchased from Adlitteram Diagnostic Laboratories (San Diego, CA, USA); MMP-3 ELISA kit was purchased from USCN Life Science Inc. (Wuhan, China); Safranin O staining kit was purchased from ScienCell Research Laboratories (Carlsbad, CA, USA); primerScript*™*RT reagent kit was purchased from TaKaRa (Dalian, China); power SYBR Green PCR Master Mix was purchased from Applied Biosystems (Carlsbad, CA, USA); phospho-STAT antibody sampler kit, phospho-c-Jun N-terminal kinases (JNK), phosphoextracellular signal-regulated kinases (ERK), phospho-p38, phospho-AKT, phospho-p65, phospho-I*κκ*B, and *β*-actin antibody were purchased from Cell Signaling Technology (Beverly, MA, USA); tissue culture reagents including Dulbecco's modified Eagle's medium (DMEM) and fetal bovine serum (FBS) were purchased from Gibco (Carlsbad, CA, USA).

### 2.2. Patients and Samples

Synovium and cartilage samples were harvested as surgical waste from 10 patients with end-stage symptomatic knee OA at the time of surgery for total knee replacement. Blood samples were collected from 30 OA patients and 30 HC. The general characteristics of both patients and controls subjects are summarized in Tables 1 and 2. OA diagnosis was determined by clinician assessment according to the American College of Rheumatology (ACR) criteria [6]. Usage of human tissues was approved by the Ethical Committee of the First Affiliated Hospital of Nanjing Medical University, and informed consent was obtained from all patients.

Blood samples were collected from peripheral veins and were processed within 1 h to provide serum for ELISA analysis and PBMC for RNA extract. Knee synovial tissues (5 OA and 3 HC samples) were used for immunohistology analysis, and the remaining 5 OA synovial samples were prepared for cell culture experiments.

### 2.3. Isolation and Culture of FLS

Primary OA FLS were isolated by enzymatic digestion of synovial tissues. In general, synovial tissue was minced and digested with 1% collagenase II at 37°C for 4 h. OA FLS were cultured in DMEM medium supplemented with 10% fetal bovine serum (FBS), 100 U/mL penicillin, and 100 *μ*g/mL streptomycin at 37°C in a humidified atmosphere of 5% CO_2_ in air. Purity of OA FLS was determined by flow cytometry stained with antifibroblast marker (FGF-2). Cells were used for further experiments if >95% cells were positive for fibroblast marker. Passages 2 to 5 cells were used in all experiments.

### 2.4. Flow Cytometry

IL-28R*α* expression on cell surface was measured using flow cytometric analysis. In brief, OA FLS (1 × 10^6^) were harvested, blocked with 1% bovine serum albumin (BSA) for 30 min, and then stained with PE-conjugated IL-28R*α* for 25 min at room temperature in the dark. After washing 3 times with PBS, positive cells were determined using a BD FACSCalibur Flow Cytometer and CellQuest software (BD Biosciences).

### 2.5. MTT Assay

Cell viability was assessed by 3-(4,5-dimethylthiazol-2-yl)-2,5-diphenyltetrazolium bromide (MTT) assay following our published procedures [15]. Briefly, OA FLS (5 × 10^3^) were incubated in a 96-well, flat-bottomed culture plate in a final volume of 200 *μ*L/well culture medium with IL-29 (1, 10, and 100 ng/mL) for 72 h in a humidified atmosphere (37°C at 5% CO_2_). Next, 20 *μ*L of cell proliferation reagent WST-1 was added to each well and incubated for a further 4 hours. After incubation, the absorbance was measured at 450 nm using an ELISA plate reader (BioTek, Winooski, VT, USA).

### 2.6. Stimulation of FLS with IL-29

OA FLS were stimulated with IL-29 (1, 10, and 100 ng/mL) in the presence or absence of IL-29 blocking antibody (1000, 2000, and 3000 ng/mL) for 48 h. After incubation, cells were collected for gene expression analysis by real-time PCR. In another set, OA FLS were treated with 100 ng/mL IL-29 for 0 min, 20 min, 40 min, and 60 min, respectively. After incubation, cells were collected for signaling pathway analysis by western blot.

### 2.7. Coculture of FLS with* Ex Vivo* Cartilage Explant

OA cartilages were obtained from knee joint tissue and submerged in a solution with betadine and PBS (1 : 3) for 10 min and then rinsed with PBS with no any trace of betadine. After freeze-thaw three times at −80°C to kill any live cell within the tissue, cartilages were stored at −20°C and ready for use.

OA FLS were cultured in 24-well plate until being 90% confluent and changed to fresh DMEM medium. Then, the cells were treated with IL-29 (1, 10, and 100 ng/mL) and IL-1*β* (20 ng/mL, positive control). Thawed cartilage tissues were diced to similar size and placed to each well including OA FLS and IL-29. Cartilage, OA FLS, and IL-29 or IL-1*β* were coincubated in DMEM medium at 37°C in a humidified incubator (5% CO_2_/air). After 72 h incubation, cartilage tissues were removed from culture wells and fixed in 10% formalin. Cell supernatants were collected and stored at −80°C for ELISA assay.

### 2.8. Real-Time PCR

Total RNA was extracted from PBMC and fibroblasts using TRIzol (Invitrogen). RNA was transcribed into cDNA in a 20 *μ*L mixture containing 1 *μ*g of total RNA and PrimeScript*™* RT Master Mix (Takara) according to the manufacturer's instructions. Quantitative real-time PCR was performed on Applied Biosystems 7900 HT Instrument (Applied Biosystems, CA, USA) following our published procedures [7]. The primer sequences were summarized in Table 3. All samples were assayed in triplicate. Relative gene expression was determined by the 2^−ΔΔCt^ method.

### 2.9. ELISA Assay

Human IL-29 and MMP-3 in serum or culture supernatants were measured by ELISA according to the manufacturer's instructions.

### 2.10. Immunohistochemistry and Safranin O-Fast Green Staining

Paraffin-embedded synovial tissues were sectioned, dewaxed, rehydrated, and stained following our published procedures [1]. The signals were developed with DAB substrate kit, with hematoxylin as counterstain. Each slide was evaluated by one of the authors under the microscopy (Nikon, Japan). Tissue sections were scored for staining of the lining on a 0 to 5 scale as reported [16]. For each section, the number of positively stained cells was counted in 20 fields. Scored data were pooled, and the mean ± SEM was calculated.

Cartilage explants were embedded in paraffin wax. After sectioning and deparaffin, the slides were stained with the Safranin O staining kit to examine the cartilage loss according to the manufacturer's instructions. In brief, the slides were stained in 0.1% Fast Green solution for 10 min, rinsed in 1% acetic acid for 15 sec, and stained in 0.1% Safranin O staining solution for 30 min. After dehydration in ethanol and clearing in Xylene, the slides were mounted and observed under microscope (Nikon, Japan). Safranin binds to glycosaminoglycan (GAG) and shows an orange-red color. Cartilage depth and GAG depletion were measured in (*μ*m) 6 random fields. Scored data were pooled, and the mean ± SEM was calculated.

### 2.11. Immunofluorescence Staining

For cells immunofluorescence staining, OA FLS were seeded on cover slips and stained following our published procedures [1]. Briefly, OA FLS were fixed in 4% paraformaldehyde for 10 min and permeabilized with 0.3% Triton X-100 in PBS for 5 min. Then, the cells were incubated with anti-human IL-28R*α* antibody overnight at 4°C. After washing, the cells were further incubated with goat anti-rabbit IgG/TRITC for 1 h in room temperature. Finally, the cells were washed and incubated with DAPI staining solution for 1-2 min and analyzed by fluorescence microscopy (Nikon, Japan). IL-28R*α* was stained in red and nuclei were stained in blue.

For tissue double immunofluorescence labeling, sections were incubated with a mixture of primary antibodies (rabbit anti-IL-29 pAb, mouse anti-CD68, or FGF-2 mAb) at 4°C overnight. After washing, the slides were further incubated with a mixture of donkey anti-rabbit IgG-R and DyLight488-conjugated donkey anti-mouse IgG for 1 h. Finally, the slides were washed and incubated with DAPI staining solution for 3–5 min. Images were acquired and processed digitally under the fluorescence microscopy (Olympus, Japan). The contained positive cells were stained in yellow and nuclei were stained in blue.

### 2.12. Western Blot Assay

For western blotting assay, cellular proteins were resolved by 8%–10% sodium dodecyl sulfate-polyacrylamide gel electrophoresis (SDS-PAGE) and were transferred to polyvinylidene fluoride membranes (Millipore, Bedford, MA, USA). Nonspecific interactions were blocked with 5% skim milk for 2 h and were then probed with phospho-STAT 1/2/3/4/5/6, phospho-AKT, phospho-p38, phospho-ERK, phospho-JNK, phospho-p65, and phospho-I*κκ*B. *β*-actin was used as a protein loading control. The signals were visualized with Super Signal West Dura chemiluminescent detection reagents following the manufacturer's directions (Thermo Fisher Scientific Inc., Rockford, USA), and protein bands were scanned and quantified with the Gel-pro Analyzer software (Bio-Rad, CA, USA). The relative quantification of target proteins was calculated by comparison of the bands density levels between samples with Image J software.

### 2.13. Statistical Analysis

All data were expressed as mean ± SEM and analyzed with Graphpad Prism 6 software (Graphpad software, La jolla, CA, USA). Differences between two groups were performed with Student's *t*-test for parametric data and Mann-Whitney *U* test for nonparametric data. A *P* value of <0.05 was considered significant.

## 3. Results

### 3.1. IL-29 and Its Receptor IL-28Ra Are Higher in PBMCs and Serum from OA Patients

**Figure 1:**
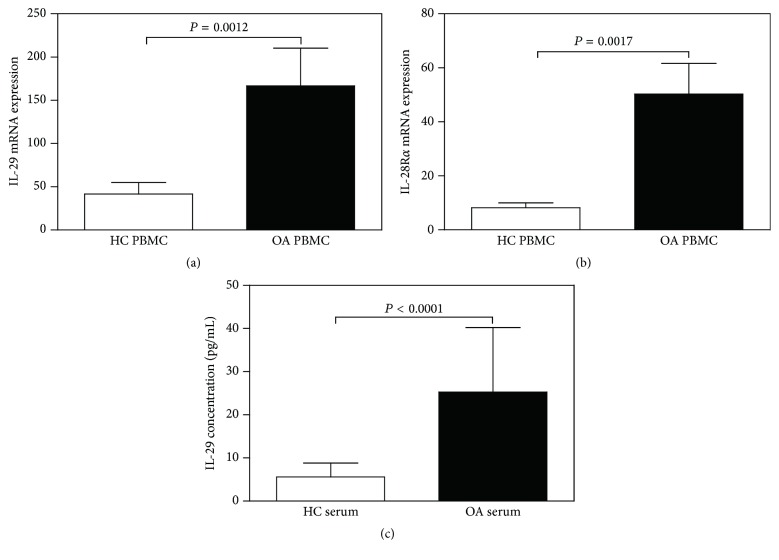
The expression of IL-29 and its receptor IL-28R*α* in the peripheral blood mononuclear cells (PBMCs) and serum of OA patients. The mRNA levels of IL-29 (a) and IL-28R*α* (b) in the OA PBMC (*n* = 30), when compared with that in HC PBMC (*n* = 30), were detected by real-time PCR. (c) The levels of IL-29 in the serum from OA patients (*n* = 30) and HC (*n* = 30) were assessed by ELISA. Data in graphs are the mean ± SEM.

To investigate whether IL-29 was involved in the pathogenesis of OA, we first examined the expression of IL-29 mRNA and its receptor IL-28Ra in PBMC. Real-time PCR analysis revealed that mRNA expression of IL-29 and IL-28Ra was significantly higher in OA PBMCs when compared to HC (*P* = 0.0012 and 0.0017, resp.; Figures 1(a) and 1(b)).

In serum, ELISA analysis showed that IL-29 was significantly higher in OA (25.26 ± 3.34 pg/mL) than in HC (5.61 ± 0.71 pg/mL, *P* < 0.0001; Figure 1(c)).

### 3.2. Both IL-29 and IL-28Ra Expression Are Elevated in Synovial Tissue from OA Patients

**Figure 2:**
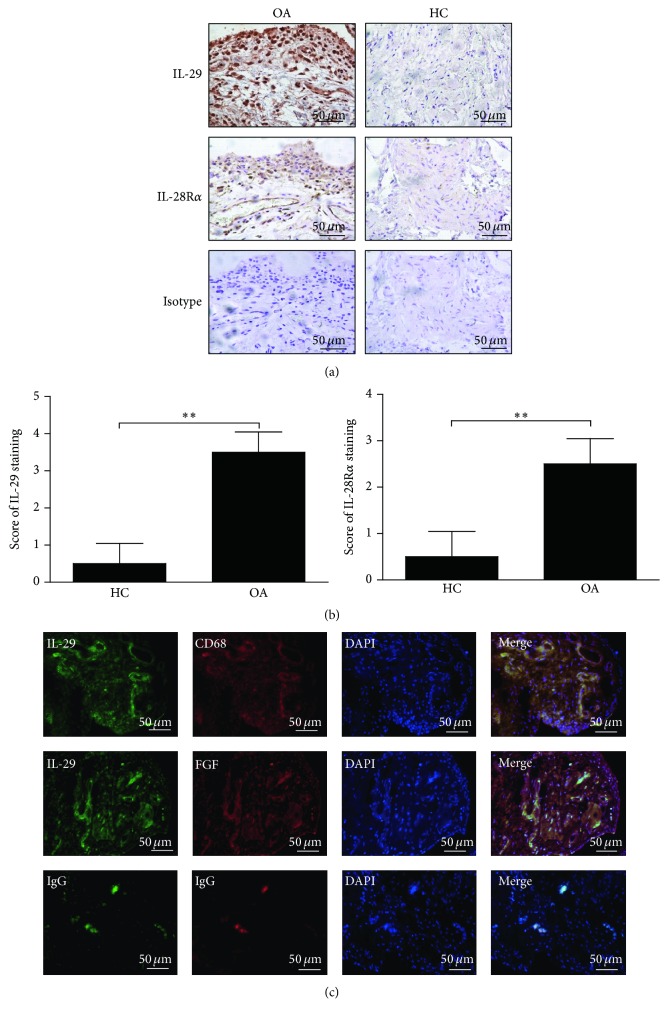
The expression and cellular distribution of IL-29 and IL-28R*α* in the synovial tissues. IL-29 and IL-28R*α* in synovial tissues of OA patients (*n* = 5) and HC (*n* = 3) were detected by immunostaining (a) and semiquantification (b). Values in (b) are the mean ± SEM. ^*∗∗*^ *P* < 0.01 versus medium control. (c) Colocalization of IL-29 with CD68 or FGF-2 in OA synovium was detected by double immunofluorescence staining, and nuclei were counterstained with DAPI. All of the magnification in this figure was ×400.

Next, we assessed the expression and localization of IL-29 and IL-28Ra in synovial tissues from 5 OA patients and 3 HC with immunohistochemical analysis. As shown in Figure 2, IL-29 and IL-28Ra are strongly expressed in the lining layer of OA synovial tissues (Figure 2(a)), and semiquantitative analysis indicated that IL-29 and IL-28Ra were significantly increased in OA synovium compared to HC (*P* = 0.0045) (Figure 2(b)).

Because macrophages and FLS are the major effector cells for inflammation in the infiltrated OA synovium [4], we next examined whether macrophages and FLS can produce IL-29 using double immunofluorescence staining. The specific antibodies against CD68 and FGF-2 served as markers for macrophages and fibroblasts, respectively. As shown in Figure 2(c), IL-29-positive cells were stained in red whereas CD68- or FGF-2-positive cells were stained in green, and positive cells for containing IL-29 with CD68 or FGF-2 were shown in yellow. As expected, IL-29 was strongly expressed in macrophages and fibroblasts in OA synovium, suggesting that these cells might be the important cellular sources of IL-29 in OA synovium.

### 3.3. IL-29 Induces Proinflammatory Mediators in OA FLS

**Figure 3:**
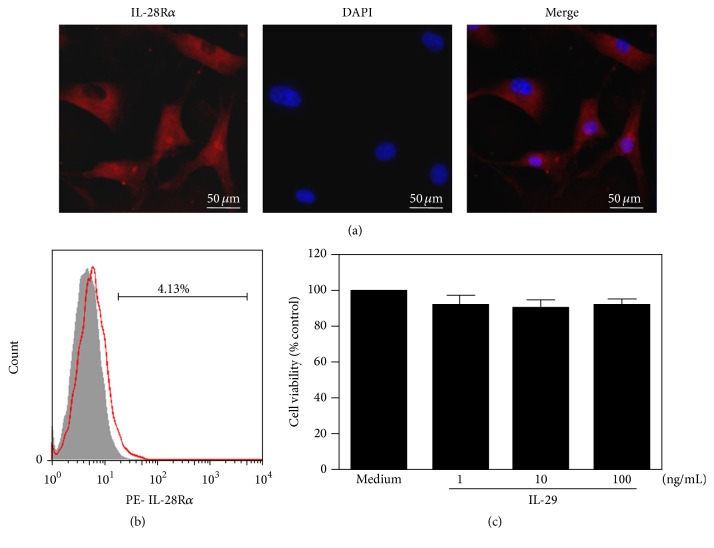
Immunostaining for IL-28R*α* in OA fibroblasts and viability of OA FLS in response to IL-29. The expression of IL-28R*α* in OA FLS, detected by immunofluorescent staining (a) and flow cytometric analysis (b). The magnification in (a) was ×400. (c) OA FLS viability after being incubated with IL-29 at 1, 10, and 100 ng/mL for 72 h was measured by MTT assay. The data present the mean ± SEM of three independent experiments.

Immunofluorescence staining showed that IL-28Ra was expressed in OA FLS (Figures 3(a) and 3(b)), implying that OA FLS may be the important target of IL-29. IL-29 at 1, 10, and 100 ng/mL did not affect the viability of OA FLS after 72 h treatment (Figure 3(c)). Therefore, these doses were chosen for further experiments.

**Figure 4:**
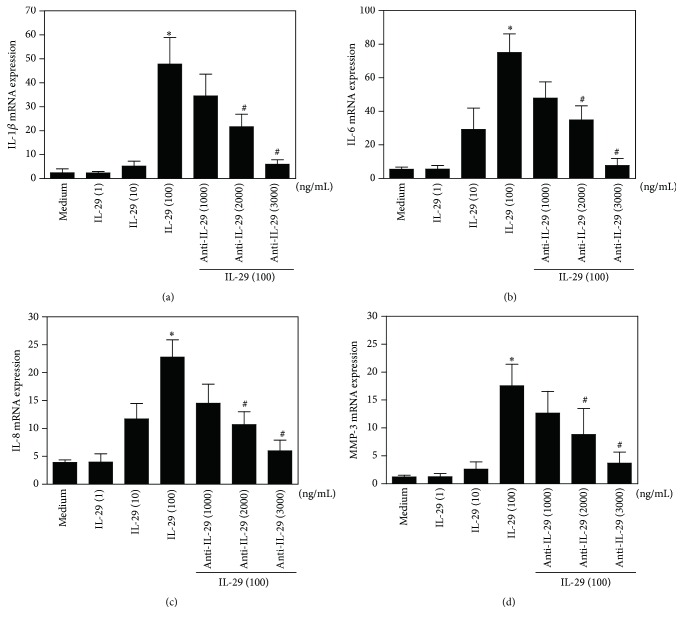
IL-29-induced cytokine expression by OA synovial fibroblasts. mRNA levels of IL-1*β* (a), IL-6 (b), IL-8 (c), and MMP-3 (d) in OA FLS after exposure to IL-29 (1, 10, and 100 ng/mL) with or without its blocking antibody for 48 h were determined by real-time PCR. The results shown are representative of one of three independent experiments. The error bars represent mean ± SEM for triplicate wells. ^*∗*^ *P* < 0.05 versus medium control. ^#^ *P* < 0.05 versus IL-29 (100 ng/mL) treatment group.

As shown in Figure 4, IL-29 induced a dose-dependent upregulation of IL-1*β*, IL-6, IL-8, and MMP-3 mRNA in OA FLS following 48 h incubation. Furthermore, the action of IL-29 on these cytokine expressions in OA FLS was abolished by addition of IL-29 blocking antibody at the concentration of 2000 and 3000 ng/mL. These data indicate that IL-29 stimulates proinflammatory cytokine expression on OA FLS.

### 3.4. IL-29 Increases Cartilage Degradation

**Figure 5:**
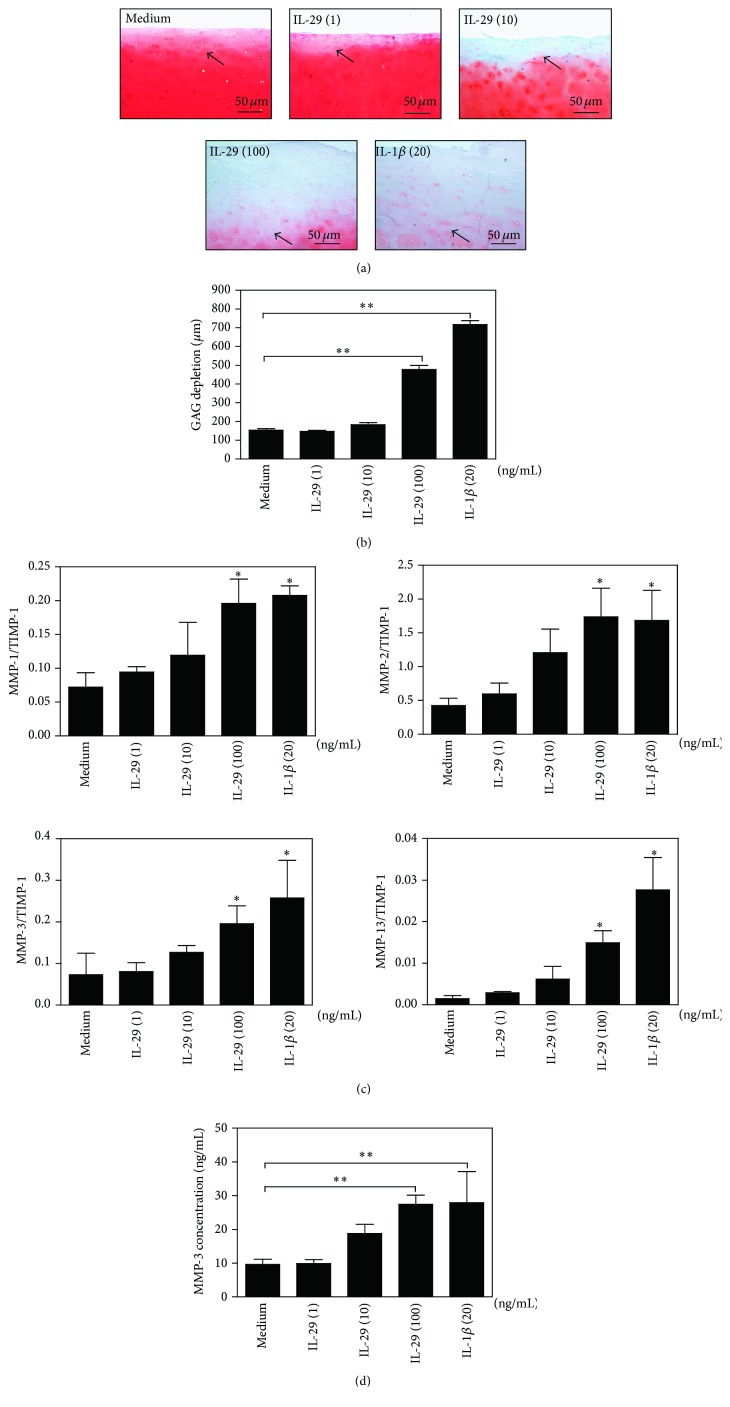
Effects of IL-29 on cartilage degradation by OA FLS* ex vivo*. (a) The OA cartilage depletion after being cocultured with OA FLS for 72 h was visualized in Safranin O/Fast Green-stained sections. Representative images from one experiment are reported (a), and the reduced intensity of red stain denotes proteoglycan loss (original magnification ×100). (b) The depth of GAG depletion (*μ*m) in cartilage was measured from the articular surface to the red/orange tidemark (marked by arrow). (c) The ratio of MM-1, MM-2, MM-3, and MM-13 to TIMP-1 was determined by real-time PCR in OA FLS. (d) MMP-3 in culture supernatants harvested from each well after 72 h was measured by ELISA. The error bars represent mean ± SEM for triplicate experiments. ^*∗*^ *P* < 0.05 versus medium control. ^*∗∗*^ *P* < 0.01 versus medium control.

IL-29 can stimulate proinflammatory cytokines expression in OA FLS as shown in Figure 4, and chronic inflammation is a major driver of ongoing cartilage damage and joint degeneration in OA pathogenesis. Therefore, we chose a coculture model of OA FLS with* ex vivo* cartilage explant to examine the influence of IL-29 on cartilage degradation. After 72 h culture, Safranin O/Fast Green staining of the cartilage in the coculture showed that glycosaminoglycan (GAG) depletion (mean ± SEM) was significantly greater in IL-29 (100 ng/mL) (477.4 ± 22.0 *μ*m) or IL-1*β* (20 ng/mL) (717.4 ± 20.9 *μ*m) treated conditions than that in the medium alone (154.4 ± 7.1 *μ*m), indicated by the less intense red staining for Safranin O and GAG depletion deeper (Figures 5(a) and 5(b)).

In OA, cartilage damage is predominantly mediated by MMPs and its specific inhibitors, the tissue inhibitors of metalloproteinases family (TIMPs), most notably by MMP-1, MMP-3, MMP-13, and TIMP-1 [14]. We examined the ratio of MMP-1, MMP-2, MMP-3, and MMP-13 to TIMP-1 in the OA FLS after 72 h coculture and found that IL-29 could significantly upregulate MMP-1/TIMP-1, MMP-2/TIMP-1, MMP-3/TIMP-1, and MMP-13/TIMP-1 ratio at a dose-dependent manner in OA FLS. We also measured MMP-3 levels in supernatant at 72 h incubation in this experiment. Consistent with our finding in OA FLS monocultures, there were higher levels of MMP-3 in OA FLS/cartilage coculture system in response to IL-29 at 100 ng/mL when compared to nontreatment control (Figure 5(c)), similar to that stimulated by IL-1*β* (20 ng/mL). These data imply that IL-29 promotes cartilage degradation by stimulating protease production in OA FLS.

### 3.5. IL-29 Induces Signal Transduction in OA FLS

**Figure 6:**
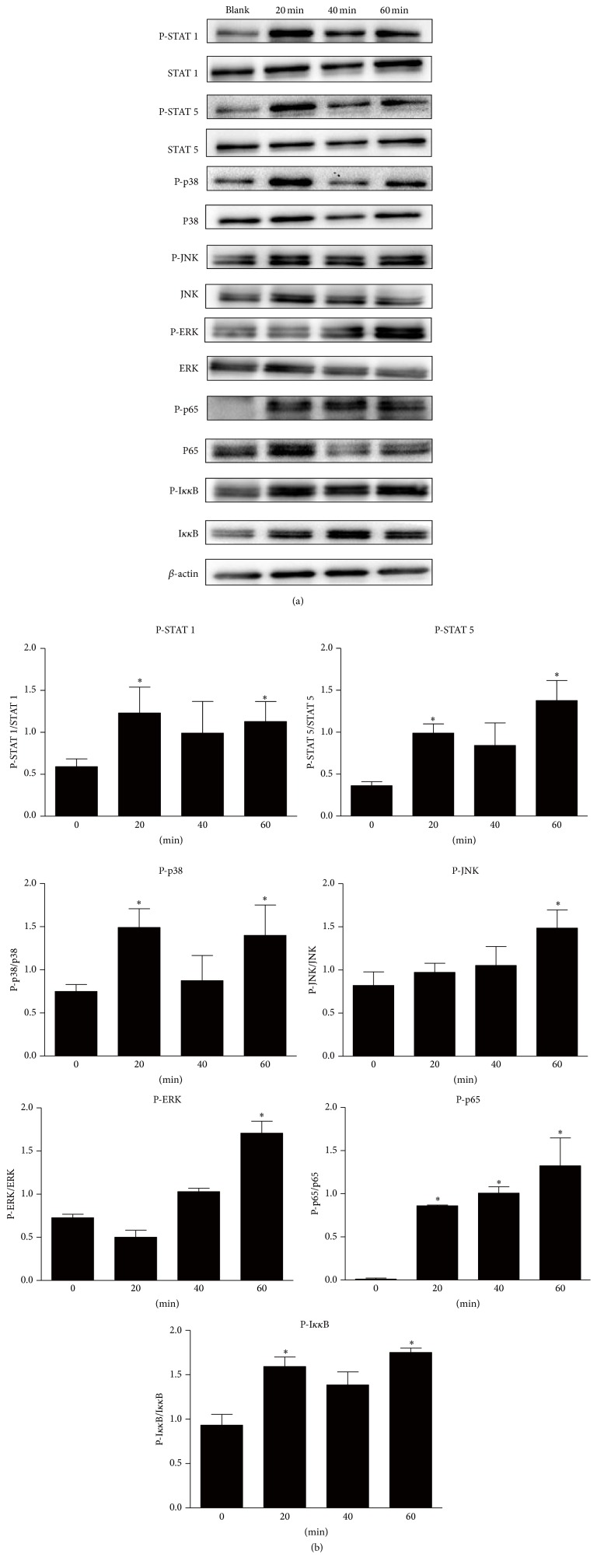
Effect of IL-29 on the phosphorylation of Jak-STAT, AKT, MAPK, and NF-*κ*B signaling pathways. OA FLS were treated with IL-29 at 100 ng/mL for 0 , 20, 40, and 60 min, and the activation of STAT 1/2/3/4/5/6, AKT, JNK, ERK, P38, p65, and NF-*κ*B was evaluated by western blot. The relative quantification of target proteins was calculated by comparison of the bands density levels between samples. The results were expressed as mean ± SEM from three independent experiments. ^*∗*^ *P* < 0.05 versus nontreatment group.

OA FLS were stimulated with IL-29 and the activation of downstream signal transduction pathways including canonical Jak-STAT and noncanonical MAPK, AKT, and NF-*κ*B pathways was evaluated (Figure 6). Following stimulation of OA FLS for 20 min, 40 min, and 60 min with IL-29 (100 ng/mL), phosphorylation of STAT 1 (Tyr701) and STAT 5 (Tyr694); JNK, ERK, and P38; and P65 and I*κκ*B was significantly increased when compared to nontreatment controls. No change in phosphorylation of STATs 2 (Tyr690), 3 (Tyr705), and 6 (Tyr641) and AKT was observed in response to IL-29. Phosphorylation of STAT 3 (Ser727) and STAT 4 (Tyr693) was not detectable in OA FLS.

## 4. Discussion

OA has traditionally been classified as a noninflammatory arthritis; however, the accumulating evidences demonstrate that joint inflammation and synovitis play a critical role in the pathogenesis of OA. Our study for the first time showed that IL-29, a novel member of type III interferon family, (i) was upregulated in blood and synovial tissues in patients with OA; (ii) enhanced inflammation of OA FLS and promoted cartilage degradation; and (iii) activated MAPK and NF-*κ*B signaling pathway.

In the first case, we demonstrate an essential role of IL-29 in inflammation in OA FLS. We found that IL-29 was abnormally elevated in PBMC, serum, synovial fluid, and synovium in OA patients. Within the joint, IL-29 and its receptor were located at FLS and macrophages, two main cell types in the inflamed synovium responsible for the inflammation and cartilage and bone damage in the joint. Abnormal IL-29 was also found in RA [1], SLE [11], gastric cancer [17], or other diseases. Similar to RA, in this study, we did not find a significant correlation between IL-29 in serum and OA disease activity (data not shown). The lack of a significant association may be due to the limited sample size and most of the patients having end-stage symptom of knee OA. Thus, further prospective studies including a larger sample size and OA patients in early and late disease are needed to determine whether IL-29 can serve as a biomarker for systemic inflammation and OA disease activity.

We have reported that IL-29 contributed to synovial inflammation by stimulating production of proinflammatory cytokines in RA FLS [1]. The proinflammatory effects of IL-29 have also been shown on PBMC by upregulation of IFN-inducible protein-10 (IP-10), monokine induced by IFN-*γ* (MIG), IFN-*γ*-inducible T cell *α* chemoattractant (I-TAC/CXCL11), and IL-8 [11, 18] and on T cells by downregulating Th2 polarization and cytokine production [15, 19, 20]. Soluble inflammatory factors such as cytokines are central to most inflammatory processes, and several cytokines have been implicated in OA pathogenesis including IL-6 and IL-8, TNF-*α*, and IL-1*β*. Indeed, our data showed that IL-29 stimulated inflammatory cytokines IL-1*β*, IL-6, and IL-8 and production in OA FLS, indicating that IL-29 may contribute to OA inflammation.

Recent epidemiological studies on large number of OA patients suggested that an inflammatory synovium/synovitis was linked to increased cartilage damage and pain [21]. FLS, the major cellular constituent of the synovial membrane, are the main contributors to the inflammation and cartilage degrading MMP overproduction within the arthritic joint [11, 12, 22]. Proinflammatory cytokines derived from FLS including IL-6 and IL-8, TNF-*α*, and IL-1*β* could increase the production of MMPs leading to cartilage damage. Our data also showed that MMP-3 mRNA was significantly increased in FLS upon IL-29 treatment; hence, we further address the interaction between IL-29, inflammation, MMP production, and cartilage degradation.

The second novel finding of this study is that IL-29 plays a critical role in cartilage degradation in OA. We used a coculture system to establish a direct link between IL-29 and cartilage degradation. In this model, FLS were cultured with OA cartilage explant to model inflammation-induced MMP production and cartilage degradation. Our data showed that IL-29 enhanced MMPs/TIMP-1 ratio in OA FLS and then resulted in marked increased GAG depletion in cartilage. Taken together, our data suggest an important role for IL-29 in regulating cartilage degradation by driving cytokine and MMP production in OA FLS. IL-29 is thus capable of modulating one of the most important processes driving tissue degeneration in OA pathogenesis.

It has been suggested that IL-29 elicits signal transduction via activation of the canonical Jak-STAT pathway and other alternative pathways including AKT and the mitogen-activated protein kinase (MAPK) in a panel of cells [23–25]. However, the ability of IL-29 to trigger the pathways could be cell-type specific or altered in cells. The present study is the first to report the signal transduction pathways in response to IL-29 in OA FLS. In these cells, IL-29 stimulated the phosphorylation of STATs 1 and 5. Phosphorylated STATs form various homodimers and heterodimers, translocate to the nucleus, and induced IL-29-specific biological activities, such as antiviral protection, antiproliferative response, and antitumor activities [26]. And the most prominent biological function of IL-29 resides in their ability to induce antiviral state in cells; thus, the canonical Jak-STAT signal pathway is the major pathway involved in IL-29-induced antiviral action, whereas the effect of IL-29 on proinflammatory cytokines production in OA FLS may be one key point for studying OA pathogenesis. It is therefore hypothesized that alternative signaling pathways could be more important in OA FLS after IL-29 stimulation. Indeed, IL-29 induced strong NF-*κ*B and MAPK (P-JNK, P-ERK, and P38) signal transduction in OA FLS. However, whether these pathways are involved in IL-29 mediated effects on OA FLS has to be investigated in the further study with the inhibitors of these pathways. Recent studies suggested that inhibitors of the above two pathways have received more attention in animal model studies and clinical trials in OA patients [12].

Collectively, our present data combined with these reports suggested that IL-29-mediated inflammation was the pivotal function of IL-29 in OA pathogenesis and inhibition of IL-29 would be a potential therapeutic target in OA disease.

## 5. Conclusions

In summary, this study provides the first evidence that IL-29 is dysregulated in OA patients and may contribute to synovial inflammation and cartilage degeneration during OA pathogenesis by production of proinflammatory cytokine. Further studies of the immunoregulatory effects of IL-29 and its underlying molecular mechanism are warranted to understand the pathological role of IL-29 in OA disease.

## Figures and Tables

**Table 1:** Demographics of patients with OA from which synovial tissues were obtained from knee replacement surgeries.

	OA	
	Female	Male
Patient number	6	4
Age (years)	64.5 ± 3.7	65.5 ± 8.8

**Table 2:** Demographics of patients with OA and healthy controls from which blood samples were collected.

	OA	HC
Patient number	30	30
Gender (female/male)	20/10	20/10
Age (years)	65.2 ± 7.9	43.2 ± 8.7

**Table 3:** Human primers used for real-time quantitative PCR.

Gene symbol	Forward (5′-3′)	Reverse (5′-3′)
IL-29	GAAGCAGTTGCGATTTAGCC	GAAGCTCGCTAGCTCCTGTG
IL-28Ra	CCTCCCCAGAATGTGACGC	GGAGCCATGTCAGGTACACG
IL-1*β*	ATGATGGCTTATTACAGTGGCAA	ATGATGGCTTATTACAGTGGCAA
IL-6	AACCTGAACCTTCCAAAGATGG	TCTGGCTTGTTCCTCACTACT
IL-8	CATACTCCAAACCTTTCCACCCC	TCAGCCCTCTTCAAAAACTTCTCCA
MMP-1	GGCTGAAAGTGACTGGGAAACC	TGCTCTTGGCAAATCTGGCGTG
MMP-2	TACAGGATCATTGGCTACACACC	GGTCACATCGCTCCAGACT
MMP-3	CAGGCTTTCCCAAGCAAATA	TTGCATTTGGGTCAAACTCC
MMP-13	ACTGAGAGGCTCCGAGAAATG	GAACCCCGCATCTTGGCTT
TIMP-1	GGGCTTCACCAAGACCTACA	TGCAGGGGATGGATAAACA
GAPDH	AGAAGGCTGGGGCTCATTTG	AGGGGCCATCCACAGTCTTC

## Abbreviations

OA: Osteoarthritis
HC: Healthy controls
IL-29: Interleukin-29
FLS: Synovial fibroblasts
PBMCs: Peripheral blood mononuclear cells
MMP-3: Matrix-metalloproteinase-3
IFNs: Interferons
rhIL-29: Recombinant human IL-29
DAPI: 40,6-Diamidine-20-phenylindole dihydrochloride.
